# A Validated, explainable machine learning–based preoperative risk model for microvascular invasion in hepatocellular carcinoma

**DOI:** 10.1007/s00423-026-04143-x

**Published:** 2026-08-27

**Authors:** Liu-Xin Zhou, Jin-Hong Cai, Chang-Ren Zhu, Tian-Ci Luo, Tian-Ming Gao, Kun-Qing Xiao, Sheng Ding, Chen Chen, Bao-Yu Wan, Hao Dong, Song-Song Fan, Dou-Sheng Bai, De-Cai Yu, Guo-Qing Jiang

**Affiliations:** 1 Department of General Surgery, Northern Jiangsu People’s Hospital Affiliated to Yangzhou University, Yangzhou, 225001 Jiangsu China; 2 Department of General Surgery, Northern Jiangsu People’s Hospital, Yangzhou, 225001 Jiangsu China; 3 Department of Thoracic Surgery, People’s Hospital of Macheng City, Affiliated Hospital of Hubei University of Science and Technology, Macheng, 438300 Hubei Province China; 4 Pathology Department, Northern Jiangsu People’s Hospital, Yangzhou, 225001 Jiangsu China; 5 Division of Hepatobiliary and Transplantation Surgery, Department of General Surgery, Nanjing Drum Tower Hospital, the Affiliated Hospital of Nanjing Medical University, Nanjing, 210008 China; 6 Department of Hepatobiliary Surgery, The Yangzhou Clinical Medical College of Xuzhou Medical University, Yangzhou, 225001 Jiangsu China

**Keywords:** Hepatocellular carcinoma, Microvascular invasion, Machine learning, Diagnostic model

## Abstract

**Purpose:**

This investigation aimed to develop and validate a diagnostic algorithm for preoperatively assessing the likelihood of microvascular invasion (MVI) in patients with hepatocellular carcinoma (HCC).

**Methods:**

Clinical and pathological information of patients with HCC who underwent curative resection was collected from two medical centers. Data from Nanjing Drum Tower Hospital were randomly split into training (80%) and internal validation (20%) cohorts, while data from Northern Jiangsu People’s Hospital were employed as an independent external validation cohort. Feature engineering was performed using recursive feature elimination (RFE) within the training cohort. Various machine learning models were applied, and their performance was evaluated through diverse metrics, such as receiver operating characteristic (ROC) curves. Additionally, the Shapley Additive Explanations (SHAP) method, together with tumor differentiation, Ki-67, and other relevant markers, were employed to enhance model interpretability and reliability.

**Results:**

Among the 1106 patients enrolled, 315 were pathologically confirmed to have MVI. RFE identified tumor diameter, alpha-fetoprotein (AFP), gamma-glutamyl transferase (GGT), and pan-immune-inflammation value (PIV) as key determinants of MVI risk in patients with HCC. With these variables, the XGBoost model reached area under the curve (AUC) values of 0.893 in the training cohort, 0.845 in the internal validation cohort, and 0.793 in the external validation cohort. Correlation analyses revealed that the risk score showed significant associations with tumor differentiation and the expression of Ki-67 proliferation index, Glypican-3 (GPC3), Cytokeratin 19 (CK19), and Vascular endothelial growth factor receptor 2 (VEGFR2).

**Conclusion:**

An XGBoost model incorporating tumor diameter, AFP, GGT, and PIV exhibited robust performance in assessing preoperative MVI among HCC patients.

**Supplementary Information:**

The online version contains supplementary material available at https://doi.org/10.1007/s00423-026-04143-x.

## Highlights

This study shows that PIV could be a valuable tool for preoperative MVI risk assessment.

An XGBoost model was established to accurately identify the likelihood of preoperative MVI.

This study explores the tumor heterogeneity reflected by the risk score.

**Supplementary Information:**

The online version contains supplementary material available at https://doi.org/10.1007/s00423-026-04143-x.

## Introduction

 Hepatocellular carcinoma (HCC) is among the most prevalent malignancies encountered in clinical practice and poses a major health burden worldwide [1]. Despite the curative potential of hepatic resection, local ablation, and liver transplantation [2], high postoperative recurrence rates—approaching 70% within five years—continue to compromise long-term outcomes [3]. Patients with HCC who have concomitant microvascular invasion (MVI) are considered high-risk for postoperative recurrence [4]. Evidence indicates that anatomical resection with surgical margins exceeding 1 cm can prolong recurrence-free survival(RFS) and overall survival(OS) in patients harboring MVI [5–6]. In contrast, percutaneous techniques such as radiofrequency ablation or microwave coagulation therapy may be suboptimal for this subgroup, potentially leading to higher recurrence and poorer survival outcomes [7–8]. Therefore, reliable preoperative assessment of MVI is essential to guide surgical strategy and improve prognosis.

The pan-immune-inflammation value (PIV) is a novel biomarker reflecting both systemic immune status and tumor-related inflammatory activity. Owing to its cost-effectiveness and ready availability in routine clinical practice, PIV has been investigated as a promising predictor of tumor recurrence and prognosis across several malignancies [9, 10]. Nevertheless, its potential association with MVI in HCC remains completely unexplored. Therefore, this study aimed to elucidate the relationship between PIV and MVI, and subsequently to develop and validate a model incorporating PIV for the preoperative assessment of MVI risk in patients with HCC.

## Methods

### Patients

Clinical and pathological data were retrospectively gathered from HCC patients treated at Northern Jiangsu People’s Hospital Affiliated to Yangzhou University (Yangzhou, China) from January 1, 2012, to December 31, 2024, and at Nanjing Drum Tower Hospital (Nanjing, China) from January 1, 2018, to December 31, 2023. Eligible patients met the following criteria: (1) underwent curative liver resection for HCC; (2) had a postoperative pathology report confirming HCC. Patients were excluded if they: (1) had postoperative pathology without definitive information regarding MVI; (2) received any prior anti-cancer therapy, including targeted or immunotherapy; (3) had previous malignancies or synchronous tumors at other locations. To protect patient privacy, all personal information has been anonymized and removed.

## Ethics statement

The study was conducted in accordance with the Declaration of Helsinki, and the requirement for individual informed consent was waived because this retrospective study utilized de-identified data. Ethical approval was obtained from the Ethics Committee of Northern Jiangsu People’s Hospital, Affiliated to Yangzhou University (Approval No. 2025ky253).

## Observational indicators

The observational indicators in this study include population characteristics such as age, sex, hypertension, diabetes, and underlying etiologies of HCC, including hepatitis B virus infection, hepatitis C virus infection, alcohol-related liver disease, schistosomiasis, autoimmune liver disease, and none [4]. Laboratory test results within 24 h after admission include blood routine tests and related inflammatory markers, such as red blood cell count (RBC), hemoglobin (HB), white blood cell count (WBC), neutrophil count (NE), lymphocyte count (LY), neutrophil-to-lymphocyte ratio (NLR), monocyte count (MO), eosinophil count (EO), basophil count (BA), platelet count (PLT), PIV, platelet-to-lymphocyte ratio (PLR), and systemic immune-inflammation index (SII) [5, 11]. Liver function indicators included albumin (ALB), total bilirubin (TBIL), direct bilirubin (DBIL), alanine aminotransferase (ALT), aspartate aminotransferase (AST), alkaline phosphatase (ALP), gamma-glutamyl transferase (GGT), albumin–bilirubin (ALBI) score and grade, and Child-Pugh score and grade. Renal function was assessed by creatinine (Cr) and blood urea nitrogen (BUN), while coagulation status was evaluated using prothrombin time (PT) and international normalized ratio (INR) [12]. Tumor markers included alpha-fetoprotein (AFP), carcinoembryonic antigen (CEA), cancer antigen 125 (CA125), cancer antigen 199 (CA199), cancer antigen 724 (CA724), cancer antigen 242 (CA242). Tumor characteristics such as Barcelona Clinic Liver Cancer (BCLC) staging, tumor diameter, and tumor number. The primary outcome was the presence of MVI. Secondary outcomes included RFS and OS. After surgery, patients were monitored every 6 months to 1 year. At Northern Jiangsu People’s Hospital Affiliated to Yangzhou University, only RFS was available, whereas at Nanjing Drum Tower Hospital, both RFS and OS were recorded. To ensure comparability of survival analysis results, patients who received postoperative adjuvant therapies such as immunotherapy or targeted therapy were excluded, and only those who underwent the same standardized postoperative management protocol were included.

## Evaluation of pathological specimens

All surgical specimens were independently observed and evaluated by a hepatopathologist with 20 years of diagnostic experience in the digestive system. The primary pathological indicators assessed included MVI, tumor differentiation, Ki-67 proliferation index (Ki-67), Cytokeratin 19 (CK19), Glypican-3 (GPC3), and Vascular endothelial growth factor receptor 2 (VEGFR2). MVI was identified by the presence of tumor cell nests in the portal vein, hepatic vein, or large capsular vessels of the surrounding hepatic tissue, which were lined by the endothelium and visible only under microscopy. MVI was further classified into three categories: M0, no MVI detected; M1, ≤ 5 MVI, occurring in the adjacent liver tissue area (≤ 1 cm from the tumor); M2, > 5 MVI, or MVI occurring in the distant liver tissue area (> 1 cm from the tumor) [13–14]. M0 was categorized as the MVI-negative group, while M1 and M2 were categorized as the MVI-positive group. Tumor differentiation was assessed using hematoxylin-eosin (H&E) staining. Immunohistochemistry markers included Ki-67, CK19, GPC3, and VEGFR2.

## Model Interpretation

The Shapley Additive Explanations (SHAP) offers a model-independent method for interpreting machine learning models, facilitating the understanding of how variations in each variable affect the model outputs. Here, the SHAP algorithm was utilized to analyze the risk assessment model, quantify feature contributions, and assess associations with MVI.

### Statistical analysis

Statistical analyses were carried out using R software (version 4.3.2). Continuous variables that did not follow a normal distribution were presented as medians with interquartile ranges [M(Q1, Q3)], and categorical variables were expressed as percentages. From Nanjing Drum Tower Hospital, 80% of patients were randomly selected as a training cohort. Within this cohort, recursive feature elimination (RFE) was employed to identify factors associated with MVI. Restricted cubic splines (RCS) analysis was used to examine the relationship between PIV and MVI. These selected factors were subsequently applied to develop several machine learning models—including logistic regression (LR), k-nearest neighbors (KNN), support vector machine (SVM), naive bayes (NB), and extreme gradient boosting (XGBoost)—to evaluate the risk of MVI. Model performance was assessed in training, internal validation, and external validation cohorts using receiver operating characteristic (ROC) and calibration curves, decision curve analysis, and classification metrics such as accuracy, F1 score, PPV, and NPV. Spearman correlation analysis was performed to examine the relationship between Ki-67 expression and MVI risk scores. Comparisons between groups were conducted using the Mann-Whitney U test for continuous variables, chi-square test for categorical variables, and Kaplan-Meier survival analysis with the log-rank test. A P-value < 0.05 was considered statistically significant.

## Results

### Baseline characteristics of patients

At Nanjing Drum Tower Hospital (Center 1), 896 patients initially underwent curative resection for HCC. Following application of the eligibility criteria, 655 cases were included in the analysis, with 176 (26.9%) confirmed to have postoperative MVI. Of these, 525 patients comprised the training cohort, while 130 were allocated to internal validation. At Northern Jiangsu People’s Hospital Affiliated to Yangzhou University (Center 2), 830 patients underwent curative resection for HCC. After screening based on the same criteria, 451 patients were included in the study, with 139 (30.8%) confirmed to have MVI. All eligible patients from this institution served as the external validation cohort. (Table 1).

**Table 1:** Baseline clinical characteristics of patients

Variable	Training cohort(*n* = 525)	Internal validation cohort(*n* = 130)	External validation cohort(*n* = 451)
Sex			
Male	427(81.33%)	17(13.08%)	99(21.95%)
Female	98(18.67%)	113(86.92%)	352(78.04%)
Age, years	60(53,67)	59(52,67)	64(55,70)
Underlying etiology			
HBV	369(70.29%)	88(67.69%)	290(64.30%)
HCV	24(4.57%)	4(3.08%)	56(12.42%)
Schisto.	16(3.05%)	11(8.46%)	12(2.66%)
AILD	-	-	2(0.44%)
ALD	-	-	1(0.22%)
None	116(22.10%)	27(20.76%)	90(19.96%)
Hypertension	160(30.48%)	48(36.92%)	162(35.92%)
Diabetes	72(13.71%)	31(23.85%)	85(18.85%)
Cirrhosis	284(54.10%)	70(53.85%)	391(86.69%)
WBC,×10⁹/L	5.00(3.90,6.20)	5.30(4.30,6.77)	5.11(4.10,6.22)
NE,×10⁹/L	2.90(2.20,3.80)	3.20(2.50,4.07)	3.19(2.32,4.23)
LY,×10⁹/L	1.40(1.10,1.80)	1.60(1.20,1.80)	1.33(0.96,1.77)
NLR	2.00(1.50,2.67)	2.02(1.61,2.76)	2.40(1.74,3.29)
MO,×10⁹/L	0.40(0.30,0.50)	0.40(0.30,0.50)	0.35(0.26,0.44)
EO,×10⁹/L	0.11(0.07,0.18)	0.11(0.07,0.17)	0.08(0.04,0.14)
BA,×10⁹/L	0.02(0.01,0.03)	0.02(0.01,0.03)	0.02(0.01,0.03)
RBC,×10¹²/L	4.45(4.08,4.82)	4.63(4.22,4.88)	4.49(4.02,4.90)
Hb, g/L	141.00(128.00,151.00)	146.50(135.00,153.00)	140.00(126.00,152.50)
HCT,%	41.20(37.90,44.30)	42.70(40.00,44.77)	-
MCV, fL	92.60(89.40,95.30)	91.85(89.80,95.20)	-
MCH, pg	31.50(30.30,32.80)	31.40(30.20,32.50)	-
MCHC, g/L	340.00(333.00,346.00)	340.00(335.00,345.00)	-
PLT,×10⁹/L	141.00(102.00,189.00)	152.00(107.00,213.25)	133.00(90.00,183.50)
RDW,%	12.80(12.40,13.50)	12.80(12.40,13.10)	-
PIV	97.09(54.98,180.43)	120.73(65.13,216.74)	111.38(55.00,193.80)
PLR	95.62(71.85,126.43)	96.46(80.00,131.33)	96.81(67.82,128.22)
SII	269.80(181.44,417.81)	327.76(194.43,460.66)	309.15(183.86,480.72)
ALT, U/L	26.70(18.50,38.80)	27.95(17.77,39.03)	30.00(19.00,44.50)
AST, U/L	27.60(21.50,37.90)	27.65(20.83,38.65)	32.00(24.00,45.05)
ALP, U/L	83.60(65.00,102.90)	76.35(63.52,99.42)	96.00(78.00,123.50)
GGT, U/L	46.70(27.70,86.40)	49.80(28.72,88.70)	58.00(33.00,107.00)
TBIL,µmol/L	13.00(10.00,17.20)	12.60(9.33,16.60)	14.40(10.30,19.70)
DBIL,µmol/L	3.60(2.70,5.00)	3.40(2.50,4.68)	5.60(4.20,8.10)
CHE, U/L	6.30(5.10,7.70)	6.90(5.70,8.35)	-
TP, g/L	66.40(63.00,70.20)	67.45(64.53,70.20)	-
ALB, g/L	40.00(37.70,41.80)	40.55(39.05,42.60)	43.10(39.10,46.00)
ALBI score	-2.68(-2.82,-2.48)	-2.75(-2.89,-2.60)	-2.90(-3.15,-2.58)
ALBI grade			
Grade1	312(59.43%)	97(74.62%)	331(73.39%)
Grade2	212(40.38%)	33(25.38%)	115(25.5%)
Grade3	1(0.19%)	0(0%)	5(1.11%)
Child-Pugh score	5.00(5.00,5.00)	5.00(5.00,5.00)	5.00(5.00,5.00)
Child–Pugh grade			
A	488(92.95%)	123(94.62%)	412(91.35%)
B	37(7.05%)	7(5.38%)	39(8.65%)
GLO, g/L	26.70(23.80,29.80)	26.30(24.22,29.17)	-
TBA,µmol/L	6.90(4.30,12.10)	6.35(3.92,10.50)	-
GLU, mmol/L	4.75(4.40,5.43)	4.70(4.34,5.47)	-
BUN, mmol/L	5.40(4.50,6.40)	5.70(4.62,6.77)	5.24(4.34,6.39)
Cr,µmol/L	63.00(56.00,72.00)	65.50(56.25,73.00)	79.00(68.00,88.00)
UA,µmol/L	320.00(264.00,384.00)	329.00(291.00,384.75)	-
TG, mmol/L	0.91(0.69,1.23)	0.97(0.70,1.35)	-
CHO, mmol/L	3.91(3.39,4.54)	3.80(3.32,4.38)	-
Ca, mmol/L	2.30(2.23,2.40)	2.33(2.24,2.38)	-
K, mmol/L	3.91(3.66,4.13)	3.88(3.61,4.11)	-
Na, mmol/L	141.20(139.70,142.50)	141.00(139.95,142.00)	-
CRP, mg/L	3.30(2.10,5.30)	3.50(2.23,6.18)	-
PT, s	11.60(11.00,12.20)	11.45(11.00,11.90)	13.30(12.40,14.70)
APTT, s	27.50(26.20,28.90)	27.95(26.52,28.90)	-
TT, s	18.40(17.60,19.40)	18.40(17.60,19.10)	-
FIB, g/L	2.50(2.00,3.00)	2.60(2.10,3.10)	-
DD, mg/L	0.32(0.18,0.67)	0.35(0.19,0.58)	-
INR	1.02(0.97,1.07)	1.01(0.96,1.05)	1.08(1.01,1.14)
AFP, ng/mL	31.60(4.30,429.10)	10.85(2.62,185.05)	14.60(3.52,262.86)
CEA, ng/mL	1.36(0.75,2.26)	1.34(0.73,2.39)	-
CA125,U/mL	8.90(5.90,14.60)	7.25(5.23,11.75)	-
CA199,U/mL	14.33(8.23,26.32)	11.91(7.77,22.42)	-
CA724,U/mL	1.55(1.40,3.33)	1.77(1.40,3.07)	-
CA242,U/mL	3.64(2.51,5.95)	3.71(2.62,6.13)	-
Tumor number	1.00(1.00,1.00)	1.00(1.00,1.00)	1.00(1.00,1.00)
tumor diameter, cm	4.00(2.50,7.00)	3.95(2.85,6.50)	4.00(2.50,6.00)
BCLC stage			
0	85(16.19%)	22(16.92%)	74(16.41%)
A	404(76.95%)	100(76.92%)	344(76.27%)
B	36(6.86%)	8(6.15%)	33(7.32%)
MVI			
M0	383(72.95%)	96(73.85%)	276(61.20%)
M1	104(19.81%)	25(19.23%)	143(31.71%)
M2	38(7.24%)	9(6.92%)	32(7.10%)

*HBV* hepatitis, *B *virus, *HCV* hepatitis, *C *virus, *ALD* alcohol-related liver disease, *AILD* autoimmune liver disease, *Schisto* schistosomiasis, *WBC* white blood cell, *NE* neutrophil, *LY* lymphocyte, *MO* monocyte, *EO* eosinophil, *BA* basophil, *NLR* neutrophil-to-lymphocyte ratio, *PLR* platelet-to-lymphocyte ratio, *PIV* pan-immune-inflammation value, *SII* systemic immune-inflammation index, *RBC* red blood cell, *Hb* hemoglobin, *HCT* hematocrit, *MCV* mean corpuscular volume, *MCH* mean corpuscular hemoglobin, *MCHC* mean corpuscular hemoglobin concentration, *PLT* platelet, *RDW *red blood cell distribution width, *ALT *alanine aminotransferase, *AST* aspartate aminotransferase, *ALP* alkaline phosphatase, *GGT* gamma-glutamyltransferase, *TBIL* total bilirubin, *DBIL* direct bilirubin, *CHE* cholinesterase, *TP* total protein, *ALB* albumin, *GLO* globulin, *TBA* total bile acid, *GLU* glucose, *BUN* blood urea nitroge, *Cr* creatinine, *UA* uric acid, *TG* triglyceride, *CHO* cholesterol, *Ca* calcium, *K *potassium, *Na* sodium, *CRP* C-reactive protein, *PT* prothrombin time, *APTT* activated partial thromboplastin time, *TT* thrombin time, *FIB* fibrinogen, *DD* D-dimer, *INR* international normalized ratio, *AFP* alpha-fetoprotein, *CEA* carcinoembryonic antigen, *CA125* carbohydrate antigen 125, *CA199* carbohydrate antigen 19 − 9, *CA724* carbohydrate antigen 724, *CA242* carbohydrate antigen 242, *ALBI* albumin-bilirubin, *MVI* microvascular invasion, M0 no MVI; M1, mild MVI; M2, severe MVI; “-” indicates data not collected

## Importance of factors in estimate MVI

**Fig. 1:**
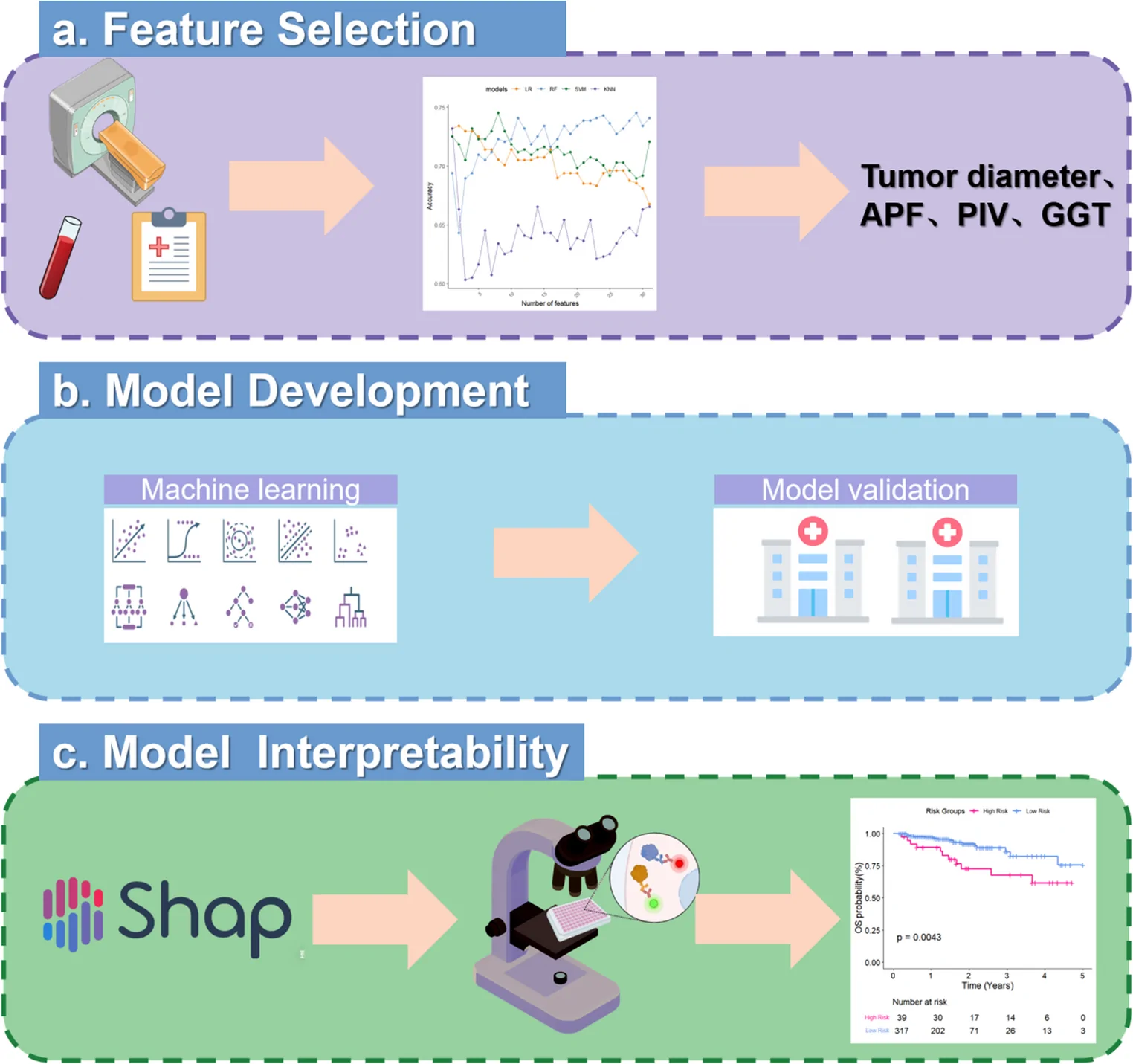
Workflow chart for the study

**Fig. 2:**
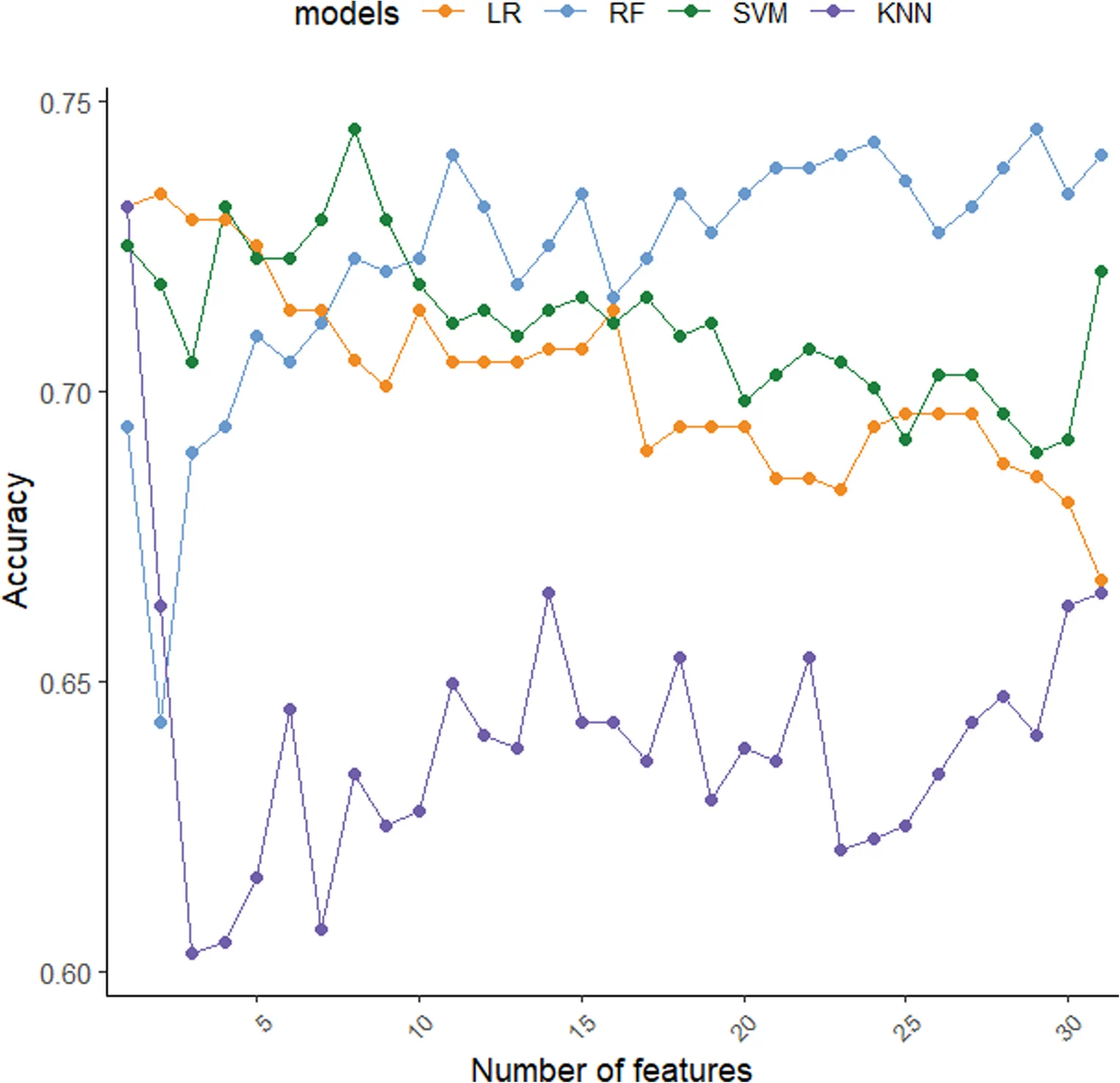
Feature selection based on Recursive Feature Elimination (RFE) using LR, SVM, RF, and KNN models

In this study, RFE was employed for variable selection. In the SVM, optimal accuracy was achieved with 8 variables, while in the RF, 11 variables yielded the highest accuracy. Although further increasing the number of variables slightly improved the model’s accuracy, we chose to retain 11 variables to maintain model simplicity (Fig. 1). To balance simplicity and clinical interpretability, we selected the intersection of the most relevant variables from both models, resulting in tumor diameter, PIV, GGT, and AFP. (Figure 2)

**Fig. 3:**
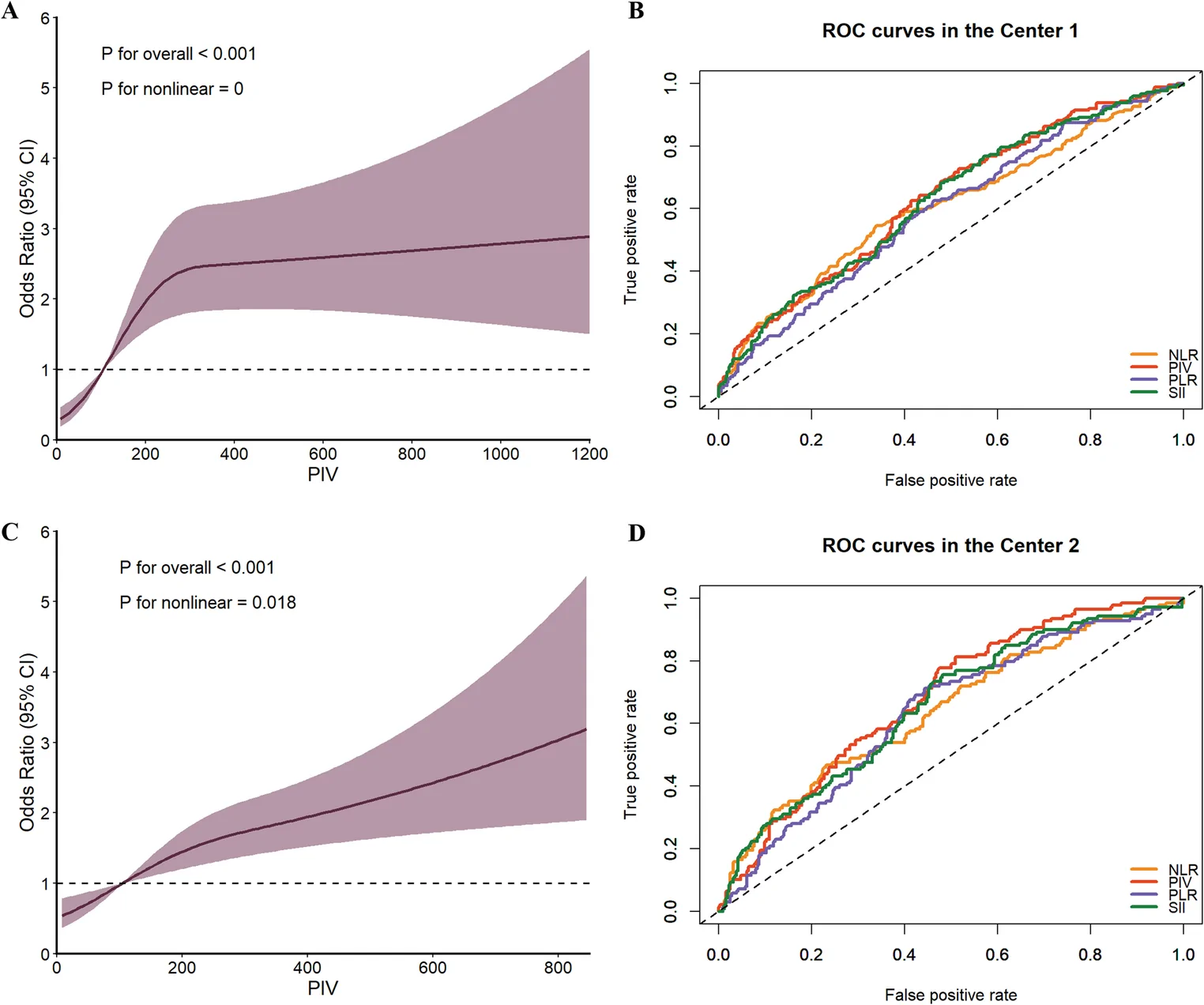
Association between PIV and MVI, and its predictive performance. (**A**, **C**) The association between PIV and MVI analyzed by restricted cubic spline regression in Center 1 (**A**) and Center 2 (**C**). (**B**, **D**) ROC curves evaluating the predictive performance for MVI in Center 1 (**B**) and Center 2 (**D**). Abbreviations: MVI, Microvascular invasion; PIV, pan-immune-inflammation value; NLR, neutrophil-to-lymphocyte ratio; PLR, platelet-to-lymphocyte ratio; SII, systemic immune-inflammation index.

In the Nanjing Drum Tower Hospital, the RCS analysis demonstrated a significant non-linear relationship between preoperative PIV and MVI (Fig. 3A). This robust correlation was consistently reproduced in the Northern Jiangsu People’s Hospital Affiliated to Yangzhou University (Fig. 3C). Furthermore, we evaluated the diagnostic superiority of PIV against other systemic inflammatory markers by ROC curves to assess the risk of preoperative MVI. In the Nanjing Drum Tower Hospital, PIV maintained its superior predictive performance with an AUC of 0.633, compared to 0.626 for SII, 0.607 for NLR, and 0.594 for PLR (Fig. 3B). Consistently, in the Northern Jiangsu People’s Hospital Affiliated to Yangzhou University, PIV achieved the highest area under the curve (AUC = 0.681), outperforming SII (AUC = 0.657), NLR (AUC = 0.643), and PLR (AUC = 0.634) (Fig. 3D).

### Performance of models

**Fig. 4:**
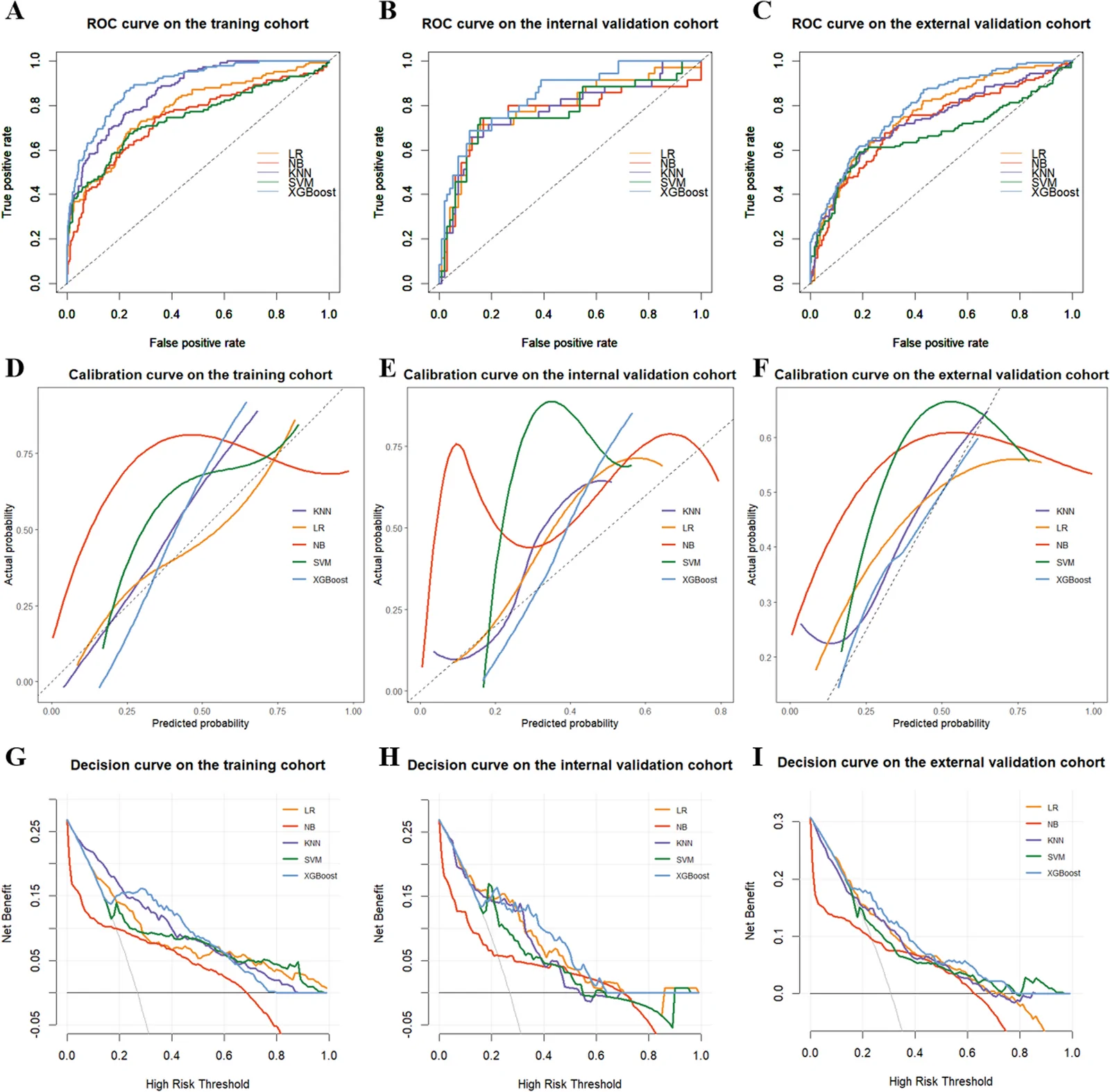
(**A**-**C**) ROC curves for different models in the training cohort (**A**), internal validation cohort (**B**), and external validation cohort (**C**). (**D**-**F**) Calibration curves for different models in the training cohort (**D**), internal validation cohort (**E**), and external validation cohort (**F**). (**G**-**I**) Decision curve analysis (DCA) for different models in the training cohort (**G**), internal validation cohort (**H**), and external validation cohort (**I**)

In this study, five models—LR, NB, SVM, KNN, and XGBoost—were constructed and compared. Of all models tested, XGBoost demonstrated superior discrimination, achieving area under the curves (AUCs) of 0.893, 0.845, and 0.793 across the training, internal, and external validation cohorts. To evaluate how well the model-derived MVI risks corresponded to real-world outcomes, calibration curves were plotted, demonstrating that the XGBoost model aligned most closely with observed events. The clinical decision curve similarly indicated that the XGBoost model provided the greatest clinical benefit across all datasets (Fig. 4). Table 2 summarizes the risk stratification outcomes and model performance across the training, internal validation, and external validation cohorts.

**Table 2:** Performance of the constructed models on training and internal/external validation sets

Model	Cohort	AUC	Sensitivity	Specificity	PPV	NPV	Accuracy	F1
LR	Training	0.782	0.376	0.935	0.679	0.803	0.785	0.484
LR	Internal Validation	0.787	0.229	0.968	0.727	0.773	0.769	0.348
LR	External Validation	0.761	0.317	0.939	0.698	0.755	0.747	0.436
NB	Training	0.740	0.319	0.943	0.672	0.790	0.775	0.433
NB	Internal Validation	0.764	0.200	0.968	0.700	0.767	0.762	0.311
NB	External Validation	0.716	0.309	0.926	0.652	0.751	0.736	0.420
KNN	Training	0.865	0.34	0.984	0.889	0.803	0.811	0.492
KNN	Internal Validation	0.777	0.086	0.979	0.600	0.744	0.738	0.150
KNN	External Validation	0.731	0.237	0.968	0.767	0.74	0.743	0.363
SVM	Training	0.746	0.362	0.979	0.864	0.807	0.813	0.510
SVM	Internal Validation	0.772	0.171	0.979	0.750	0.762	0.762	0.279
SVM	External Validation	0.667	0.273	0.946	0.691	0.745	0.738	0.392
XGBoost	Training	0.893	0.376	0.982	0.883	0.811	0.819	0.527
XGBoost	Internal Validation	0.845	0.286	0.979	0.833	0.788	0.792	0.426
XGBoost	External Validation	0.793	0.237	0.971	0.786	0.741	0.745	0.365

*LR* logistic regression, *DT* decision tree, *KNN* k-nearest neighbours, *NB* naive Bayes,* XGBoost* extreme gradient boosting, *AUC* area under the curve, *PPV* positive predictive value, *NPV* negative predictive value, *TPR* true positive rate, *TNR* true negative rate, *ACC* accuracy

### Model explanation

**Fig. 5:**
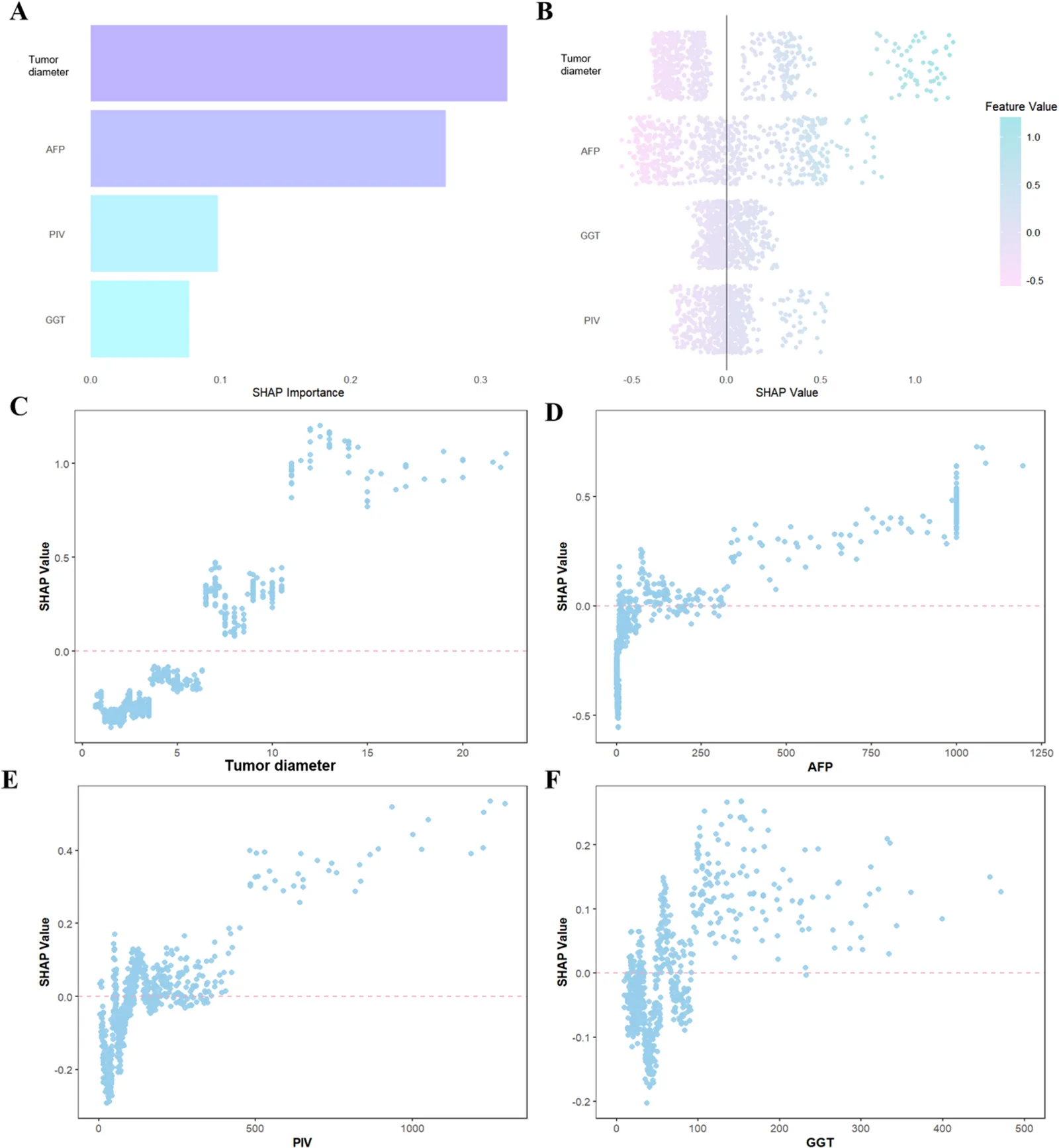
Global model explanation based on the Shapley Additive Explanations (SHAP) method. (**A**) SHAP summary bar plot, showing the importance ranking of different features in the model. (**B**) SHAP beeswarm plot, illustrating the relationship between the SHAP values of each feature and the occurrence of preoperative MVI. Each point represents the SHAP value for each patient in the model, and the color of the points indicates the actual value of the feature for the patient. The points are stacked vertically to show density. (**C** - **F**) SHAP dependence plot, demonstrating how each feature affects the output of the prediction model. Each point represents a patient, and higher SHAP values for specific features indicate a higher risk of preoperative MVI. Abbreviations: MVI, Microvascular invasion; AFP, Alpha-fetoprotein; PIV: Platelet-to-lymphocyte ratio; GGT, Gamma-glutamyl transferase

Given that machine learning models have a “black-box” effect and their reasoning process cannot be directly observed, we employed SHAP for model interpretation. Figure 5A displays the importance of four features in the model, where tumor diameter has the greatest importance in predicting MVI, followed by AFP, PIV, and GGT. Figure 5B demonstrates a positive association between MVI risk and tumor diameter, AFP, PIV, and GGT. Figure 5C and F further depict the individual effects of tumor diameter, AFP, PIV, and GGT on the model’s predicted risk.

### Clinical significance of the risk score

**Fig. 6:**
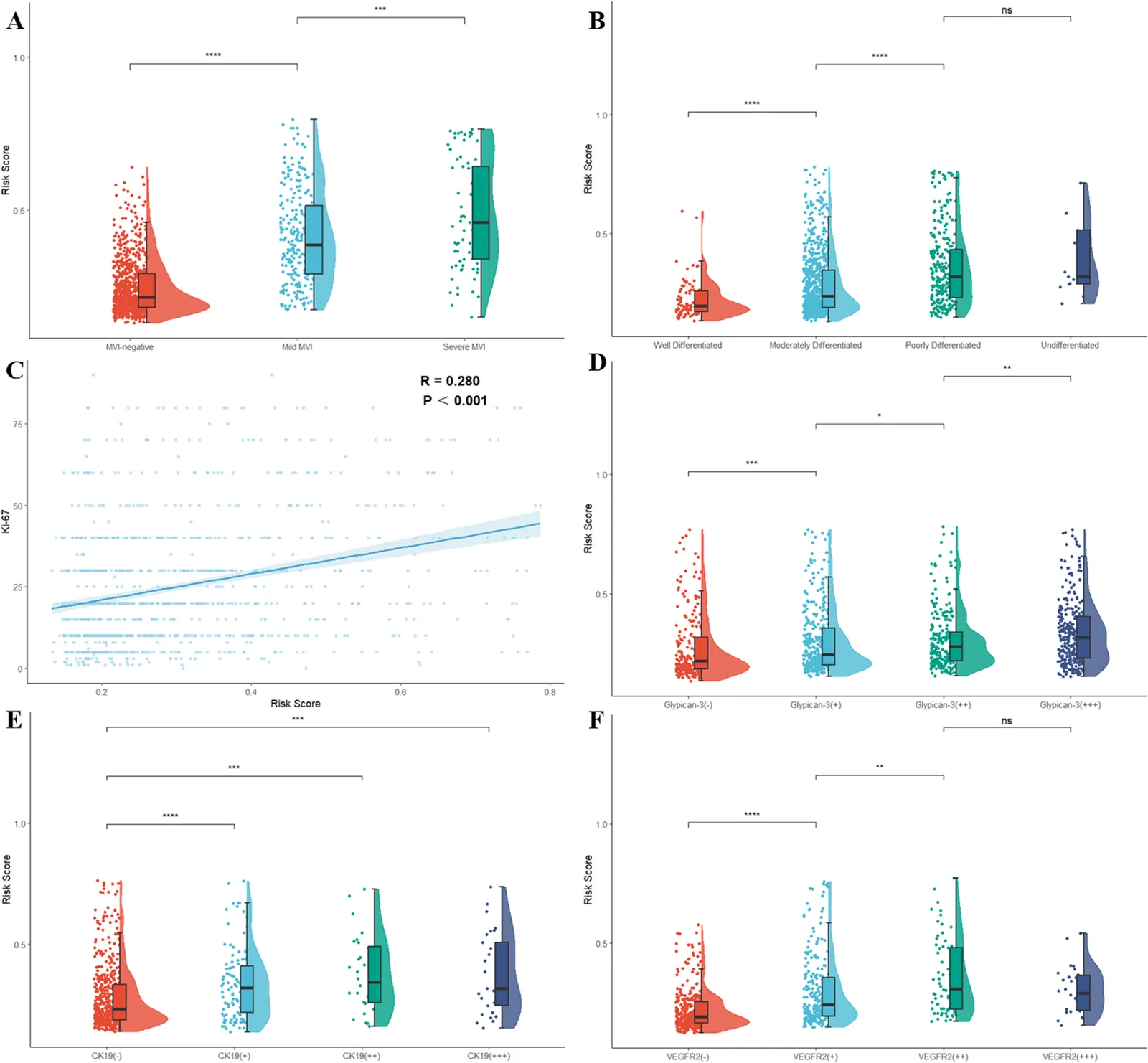
Association between the risk score and tumor pathological characteristics. (**A**) Risk score distribution across different MVI stages. (**B**) Risk score distribution across different tumor differentiation grades. (**C**) Correlation between Ki-67 expression and risk score. (D-F) Risk score distribution according to the expression levels of immunohistochemical markers: GPC3(D), CK19(E), and VEGFR2(F)

**Fig. 7:**
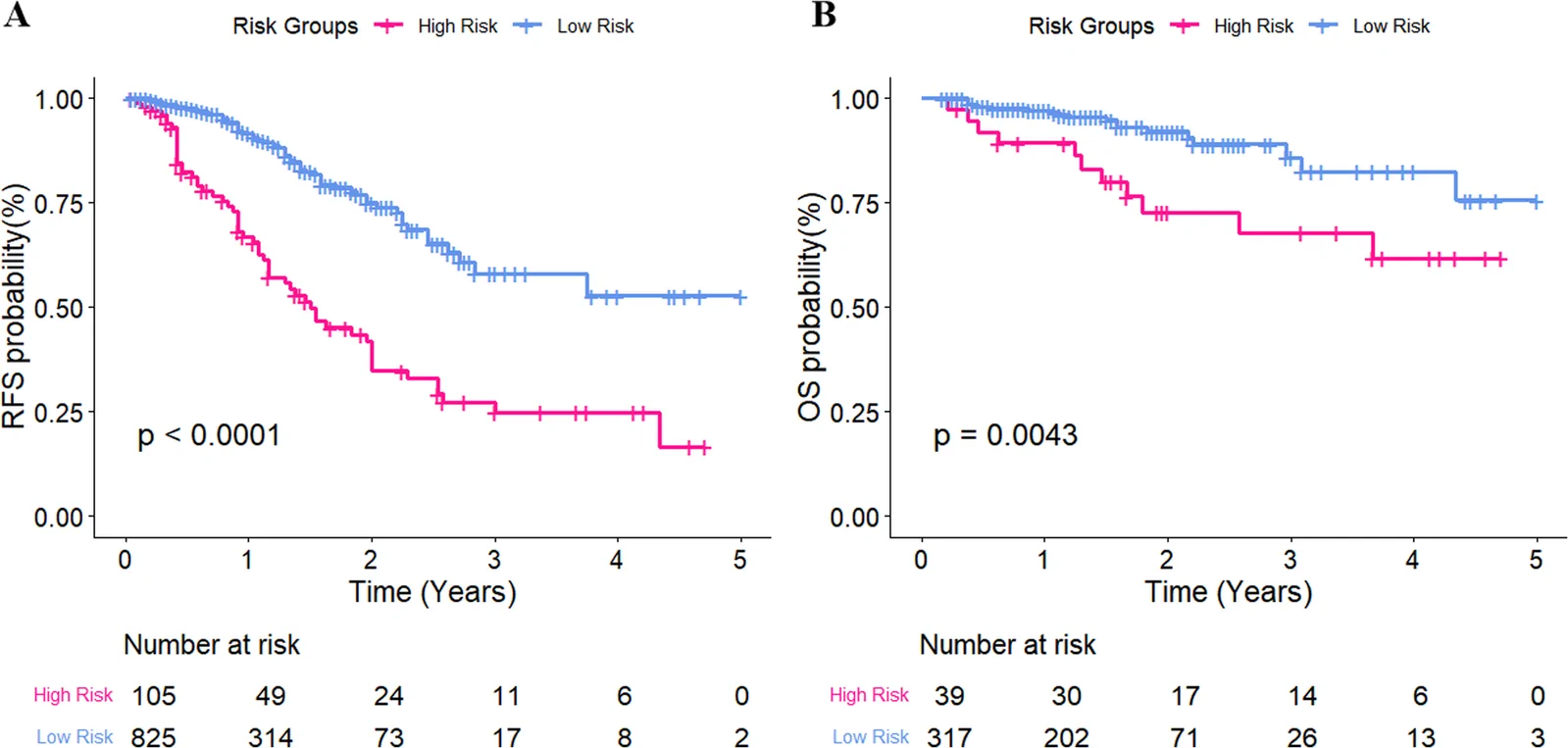
Kaplan-Meier survival curves for the high-risk and low-risk groups. (**A**) Recurrence-free survival (RFS) Kaplan-Meier curve comparing the high-risk group with the low-risk group. (**B**) Overall survival (OS) Kaplan-Meier curve comparing the high-risk group with the low-risk group

To explore the relationship between the model and tumor heterogeneity, we evaluated the association of the XGBoost model with pathological features. The results suggested that the risk score correlates with MVI severity, tumor heterogeneity, and prognosis. Regarding MVI severity, patients in the M2 group had significantly higher risk scores than those in the M1 group, and patients in the M1 group had significantly higher risk scores than those in the M0 group (*P* < 0.05). In terms of tumor heterogeneity, the risk score was significantly higher in poorly differentiated tumors than in moderately differentiated tumors, and significantly higher in moderately differentiated tumors than in well-differentiated tumors (*P* < 0.05). For GPC3 expression, patients with GPC3(+++) had significantly higher risk scores than those with GPC3(++), patients with GPC3(++) had higher scores than those with GPC3(+), and patients with GPC3(+) had higher scores than those with GPC3(-) (*P* < 0.05). Similarly, patients with VEGFR2(++) had significantly higher risk scores than those with VEGFR2(+), and patients with VEGFR2(+) had higher scores than those with VEGFR2(-) (*P* < 0.05). Patients positive for Cytokeratin 19 (CK19), including CK19(+), CK19(++), and CK19(+++) subgroups, had significantly higher risk scores than CK19-negative patients (*P* < 0.05). In addition, the risk score was positively correlated with Ki-67 expression in tumor tissues (*R* = 0.28, *P* < 0.05) (Fig. 6). Finally, patients with high-risk MVI exhibited significantly shorter RFS and OS compared with low-risk patients (*P* < 0.05) (Fig. 7).

## Discussion

The evaluation of MVI status is essential for selecting optimal therapeutic options in HCC. This study constructed a diagnostic scoring model based on commonly used clinical indicators to preoperatively assess the risk of MVI. We observed MVI incidence rates of 26.9% and 30.8% at the two centers, consistent with previously reported values [15]. During the feature selection process, in addition to the three known key factors—tumor diameter, AFP, and GGT—that are used to assess MVI, this study also identified the potential value of the PIV in assessing preoperative MVI risk [16–18]. We hypothesize that elevated PIV may reflect the remodeling of the tumor microenvironment, immune suppression, and the enrichment of growth factors in an inflammatory state, which may promote MVI formation [19, 20].

Our investigation compared five prevalent machine learning approaches, revealing that XGBoost achieved the highest performance regarding both model fit and generalizability. Therefore, based on the previously established predictors (tumor diameter, AFP, and GGT) alongside the novel biomarker validated in this study (PIV), we utilized XGBoost to construct the final predictive model for preoperative MVI risk. To strengthen confidence in the model, we utilized SHAP to estimate the relative importance of each variable and to elucidate the model’s decision-making process. The results indicated that tumor diameter, AFP, PIV, and GGT were not linearly related to MVI risk, which further confirmed the rationale for choosing the XGBoost model. In addition, the beeswarm plot generated by SHAP illustrated the importance and directionality of each feature at the individual level, providing clinicians with an inference pathway, which helped enhance trust in the prediction results. To further explore the potential associations between the model and biological characteristics, we subsequently conducted an exploratory analysis. It was revealed that the risk score exhibited a relationship with MVI severity, as higher scores were linked to an increased likelihood of severe MVI. Furthermore, patients with lower tumor differentiation showed higher risk scores, suggesting that the scoring model can partially reflect the biological malignancy of the tumor. Ki-67, a protein associated with cell proliferation, is present during the cell cycle and is commonly used to indicate tumor cell proliferative activity [21]. We evaluated the correlation between the risk score and Ki-67 expression and found a significant positive correlation, suggesting that rapid tumor proliferation may promote the development of MVI. GPC3, by modulating Wnt, Hedgehog, and FGF signaling, enhances the aggressive behavior of HCC cells, thereby elevating MVI risk [22–26]. In this study, upon categorizing patients based on GPC3 levels, those in the high-expression group showed notably higher risk scores than their low-expression counterparts. CK19, an epithelial cell intermediate filament protein usually expressed in bile duct cells, serves as a marker for HCC derived from hepatic progenitor cells and indicates a more aggressive tumor phenotype [27–29]. CK19-positive individuals exhibited markedly elevated risk scores compared with CK19-negative counterparts, further supporting the model’s ability to reflect tumor invasiveness. VEGFR-2, a critical molecule in the VEGF pathway, activates Ras/Raf/MEK/ERK and other downstream signaling cascades via VEGF-A, thereby promoting angiogenesis and MVI formation [30–32]. This study further implied that VEGFR-2 levels in tumor tissues were closely associated with the risk score. In conclusion, the risk score model not only effectively assesses the risk of MVI but is also correlated with tumor differentiation, proliferative capacity, and invasiveness.

Several limitations should be acknowledged. For instance, although the cohort included individuals with non-alcoholic fatty liver disease (NAFLD), this etiology was not specifically categorized or analyzed, reflecting its recent emergence in clinical practice. Therefore, the applicability of the model to patients with NAFLD has not been independently evaluated. Second, regarding the comparison with previously published MVI prediction markers, we compared our findings without reconstructing these established models based on original coefficients, which represents a limitation of this study. Furthermore, the model was only evaluated for MVI risk in HCC patients, and its applicability to intrahepatic cholangiocarcinoma patients remains unverified. Finally, as this analysis was retrospective, it is necessary to incorporate prospective data in future studies to verify the robustness of the model.

## Conclusions

These clinical indicators—tumor diameter, AFP, PIV, and GGT—play a critical role in preoperatively assessing microvascular invasion in HCC patients. An XGBoost model built on these features serves as a reliable scoring system to categorize patients based on their preoperative MVI risk. 

## Supplementary Information

Below is the link to the electronic supplementary material.


Supplementary Material 1


## Data Availability

Due to ethical restrictions and the involvement of human participants, the raw data supporting the findings of this study cannot be made publicly available. The data contain sensitive information and are subject to institutional and legal confidentiality requirements. Data may be made available from the corresponding author upon reasonable request and with approval from the institutional Ethics Committee.

## References

[1] Sung H, Ferlay J, Siegel RL et al (2021) Global Cancer Statistics 2020: GLOBOCAN Estimates of Incidence and Mortality Worldwide for 36 Cancers in 185 Countries. CA Cancer J Clin 71(3):209–249. 10.3322/caac.21660 33538338

[2] Birgin E, Hetjens S, Tam M et al (2023) Stereotactic Body Radiation Therapy versus Surgical Resection for Stage I/II Hepatocellular Carcinoma. Cancers (Basel) 15(8):2330. 10.3390/cancers15082330 37190258 PMC10136632

[3] Roayaie S, Obeidat K, Sposito C et al (2013) Resection of hepatocellular cancer ≤ 2 cm: results from two Western centers. Hepatology 57(4):1426–1435. 10.1002/hep.25832 22576353 PMC3442120

[4] Rodríguez-Perálvarez M, Luong TV, Andreana L et al (2013) A systematic review of microvascular invasion in hepatocellular carcinoma: diagnostic and prognostic variability. Ann Surg Oncol 20(1):325–339. 10.1245/s10434-012-2513-1 23149850

[5] Zhou Q, Zhou C, Yin Y et al (2021) Development and validation of a nomogram combining hematological and imaging features for preoperative prediction of microvascular invasion in hepatocellular carcinoma patients. Ann Transl Med 9(5):402. 10.21037/atm-20-4695 33842623 PMC8033313

[6] Liu Y, Dai Y, Zhang XX et al (2018) [Comparative analysis of anatomic and non-anatomic hepatectomy for single small hepatocellular carcinoma with microvascular invasion]. Zhonghua Yi Xue Za Zhi 98(24):1937–1940 Chinese. 10.3760/cma.j.issn.0376-2491.2018.24.009 29996286

[7] Zheng Z, Guan R, Jianxi W et al (2022) Microvascular Invasion in Hepatocellular Carcinoma: A Review of Its Definition, Clinical Significance, and Comprehensive Management. J Oncol 2022:9567041. 10.1155/2022/9567041 35401743 PMC8986383

[8] Yamashita YI, Imai K, Yusa T et al (2018) Microvascular invasion of single small hepatocellular carcinoma ≤ 3 cm: Predictors and optimal treatments. Ann Gastroenterol Surg 2(3):197–203. 10.1002/ags3.12057 29863190 PMC5980603

[9] Ligorio F, Fucà G, Zattarin E et al (2021) The Pan-Immune-Inflammation-Value Predicts the Survival of Patients with Human Epidermal Growth Factor Receptor 2 (HER2)-Positive Advanced Breast Cancer Treated with First-Line Taxane-Trastuzumab-Pertuzumab. Cancers (Basel) 13(8):1964. 10.3390/cancers13081964 33921727 PMC8073809

[10] Zeng R, Liu F, Fang C et al (2021) PIV and PILE Score at Baseline Predict Clinical Outcome of Anti-PD-1/PD-L1 Inhibitor Combined With Chemotherapy in Extensive-Stage Small Cell Lung Cancer Patients. Front Immunol 12:724443. 10.3389/fimmu.2021.724443 34777341 PMC8586214

[11] Wang J, Chen Z, Wang L et al (2022) A new model based inflammatory index and tumor burden score (TBS) to predict the recurrence of hepatocellular carcinoma (HCC) after liver resection. Sci Rep 12(1):8670. 10.1038/s41598-022-12518-5 35606395 PMC9126887

[12] Johnson PJ, Berhane S, Kagebayashi C et al (2015) Assessment of liver function in patients with hepatocellular carcinoma: a new evidence-based approach-the ALBI grade. J Clin Oncol 33(6):550–558. 10.1200/JCO.2014.57.9151 25512453 PMC4322258

[13] Sheng X, Ji Y, Ren GP et al (2020) A standardized pathological proposal for evaluating microvascular invasion of hepatocellular carcinoma: a multicenter study by LCPGC. Hepatol Int 14(6):1034–1047. 10.1007/s12072-020-10111-4 33369707

[14] Cong WM, Bu H, Chen J et al (2016) Practice guidelines for the pathological diagnosis of primary liver cancer: 2015 update. World J Gastroenterol 22(42):9279–9287. 10.3748/wjg.v22.i42.9279 27895416 PMC5107692

[15] Lv K, Cao X, Du P et al (2022) Radiomics for the detection of microvascular invasion in hepatocellular carcinoma. World J Gastroenterol 28(20):2176–2183. 10.3748/wjg.v28.i20.2176 35721882 PMC9157623

[16] McHugh PP, Gilbert J, Vera S et al (2010) Alpha-fetoprotein and tumour size are associated with microvascular invasion in explanted livers of patients undergoing transplantation with hepatocellular carcinoma. HPB (Oxford) 12(1):56–61. 10.1111/j.1477-2574.2009.00128.x 20495646 PMC2814405

[17] Zhang H, Zhou Y, Li Y et al (2020) Predictive value of gamma-glutamyl transpeptidase to lymphocyte count ratio in hepatocellular carcinoma patients with microvascular invasion. BMC Cancer 20(1):132. 10.1186/s12885-020-6628-7 32070301 PMC7029459

[18] Wang Y, Meng B, Wang X et al (2023) Noninvasive urinary protein signatures combined clinical information associated with microvascular invasion risk in HCC patients. BMC Med 21(1):481. 10.1186/s12916-023-03137-6 38049860 PMC10696877

[19] Chen Y, Zeng J, Guo P et al (2021) Prognostic Significance of Platelet-to-Lymphocyte Ratio (PLR) in Extrahepatic Metastasis of Hepatocellular Carcinoma After Curative Resection. Cancer Manag Res 13:1395–1405. 10.2147/CMAR.S290738 33603483 PMC7886383

[20] Park Y, Chang AR (2022) Neutrophil to Lymphocyte Ratio and Platelet to Lymphocyte Ratio in Hepatocellular Carcinoma Treated with Stereotactic Body Radiotherapy. Korean J Gastroenterol 79(6):252–259. 10.4166/kjg.2022.021 35746839 PMC12286552

[21] Yang X, Ni H, Lu Z et al (2023) Mesenchymal circulating tumor cells and Ki67: their mutual correlation and prognostic implications in hepatocellular carcinoma. BMC Cancer 23(1):10. 10.1186/s12885-023-10503-3 36600214 PMC9814317

[22] Pez F, Lopez A, Kim M et al (2013) Wnt signaling and hepatocarcinogenesis: molecular targets for the development of innovative anticancer drugs. J Hepatol 59(5):1107–1117. 10.1016/j.jhep.2013.07.001 23835194

[23] Capurro MI, Xiang YY, Lobe C et al (2005) Glypican-3 promotes the growth of hepatocellular carcinoma by stimulating canonical Wnt signaling. Cancer Res 65(14):6245–6254. 10.1158/0008-5472 16024626

[24] Wang S, Chen N, Chen Y et al (2018) Elevated GPC3 level promotes cell proliferation in liver cancer. Oncol Lett 16(1):970–976. 10.3892/ol.2018.8754 29963171 PMC6019913

[25] Sun CK, Chua MS, He J et al (2011) Suppression of glypican 3 inhibits growth of hepatocellular carcinoma cells through up-regulation of TGF-β2. Neoplasia 13(8):735–747. 10.1593/neo.11664 21847365 PMC3156664

[26] Kaposi-Novak P, Lee JS, Gòmez-Quiroz L et al (2006) Met-regulated expression signature defines a subset of human hepatocellular carcinomas with poor prognosis and aggressive phenotype. J Clin Invest 116(6):1582–1595. 10.1172/JCI27236 16710476 PMC1462944

[27] Shuyao W, Mingyang B, Feifei M et al (2022) CK19 Predicts Recurrence and Prognosis of HBV Positive HCC. J Gastrointest Surg 26(2):341–351. 10.1007/s11605-021-05107-w 34506016

[28] Chu T, Zhao C, Zhang J et al (2022) Application of a Convolutional Neural Network for Multitask Learning to Simultaneously Predict Microvascular Invasion and Vessels that Encapsulate Tumor Clusters in Hepatocellular Carcinoma. Ann Surg Oncol 29(11):6774–6783. 10.1245/s10434-022-12000-6 35754067 PMC9492610

[29] Lee CW, Lin SE, Tsai HI et al (2018) Cadherin 17 is related to recurrence and poor prognosis of cytokeratin 19-positive hepatocellular carcinoma. Oncol Lett 15(1):559–567. 10.3892/ol.2017.7320 29387234 PMC5768062

[30] Chen Q, Liu Y, Jeong HW et al (2019) Apelin+ Endothelial Niche Cells Control Hematopoiesis and Mediate Vascular Regeneration after Myeloablative Injury. Cell Stem Cell 25(6):768–783e6. 10.1016/j.stem.2019.10.006 31761723 PMC6900750

[31] Tsai ML, Lee CH, Huang LC et al (2022) CRISPR-mediated knockout of VEGFR2/KDR inhibits cell growth in a squamous thyroid cancer cell line. FEBS Open Bio 12(5):993–1005. 10.1002/2211-5463.13399 35313079 PMC9063427

[32] Wang X, Bove AM, Simone G et al (2020) Molecular Bases of VEGFR-2-Mediated Physiological Function and Pathological Role. Front Cell Dev Biol 8:599281. 10.3389/fcell.2020.599281 33304904 PMC7701214

