# Supplementary material for: A Validated, explainable machine learning–based preoperative risk model for microvascular invasion in hepatocellular carcinoma
Source: Langenbecks Arch Surg. 2026 Aug 27;411(1):220. doi: 10.1007/s00423-026-04143-x (PMC13525009; doi:10.1007/s00423-026-04143-x)
Supplement: Supplementary file 1 [file 423_2026_4143_MOESM1_ESM.doc]

**A Validated, Explainable Machine Learning–Based Preoperative Risk Model for Microvascular Invasion in Hepatocellular Carcinoma**

**Liu-Xin Zhou^1,2#^, Jin-Hong Cai^3#^, Chang-Ren Zhu^4#^, Tian-Ci Luo^5^, Tian-Ming Gao^1,2^, Kun-Qing Xiao^1,2^, Sheng Ding^1,2^, Chen Chen^1,2^, Bao-Yu Wan^1,6^, Hao Dong^1,2^, Song-Song Fan^1,2^, Dou-Sheng Bai^1,2^, De-Cai Yu^5*^, Guo-Qing Jiang^1,2*^**

^1^Department of Hepatobiliary Surgery, Northern Jiangsu People's Hospital Affiliated to Yangzhou University, Yangzhou , Jiangsu 225001, China

^2^Department of Hepatobiliary Surgery, Northern Jiangsu People's Hospital, Yangzhou , Jiangsu 225001, China

^3^Department of Thoracic Surgery, People's Hospital of Macheng City, Affiliated Hospital of Hubei University of Science and Technology, Macheng, Hubei Province 438300, China

^4^Pathology Department, Northern Jiangsu People's Hospital, Yangzhou , Jiangsu 225001, China

^5^Division of Hepatobiliary and Transplantation Surgery, Department of General Surgery, Nanjing Drum Tower Hospital, the Affiliated Hospital of Nanjing Medical University, Nanjing 210008, China

^6^Department of Hepatobiliary Surgery, The Yangzhou Clinical Medical College of Xuzhou Medical University, Yangzhou , Jiangsu 225001, China

*** For correspondence:**

**Guo-Qing Jiang,** Email: jgqing2003@hotmail.com, Tel. +86-87373012.

**De-Cai Yu,** Email: [yudecai@nju.edu.cn](mailto:yudecai@nju.edu.cn), Tel. 8625-8310-5892.

\# The three authors (Liu-Xin Zhou, Jin-Hong Cai and Chang-Ren Zhu) contributed equally to this work.

**TABLE OF CONTENTS**

[Supplementary Methods	3](#_Toc8785)

[Supplementary Results	6](#_Toc4887)

[Supplementary References	10](#_Toc22998)

# Supplementary Methods

Sample Size Calculation
In this study, we calculated the minimum required sample size using the

$$\text{n}=\left(\frac{1.96}{\text{δ}}\right)^{2}\varphi^{\wedge} \left(1-\varphi^{\wedge} \right)$$

for prediction model development. Assuming a C-statistic of 0.75, an event rate of 0.3, and the inclusion of 4 predictor variables, the minimum required sample size was calculated to be 323, including 97 events. The sample size used for model development in this study was 525, with 141 events, which meets the required sample size for reliable model building.

# **Inflammatory and Liver Function Indices**

PIV(Pan-Immune-Inflammation Value) = Platelet count×Neutrophil count×Monocyte count / Lymphocyte count

SII(Systemic Immune-Inflammation Index) = Platelet count×Neutrophil count / Lymphocyte count

NLR (Neutrophil-to-Lymphocyte Ratio) = Neutrophil count / Lymphocyte count

PLR (Platelet-to-Lymphocyte Ratio) = Platelet count / Lymphocyte count

ALBI Score = (log10 bilirubin × 0.66) + (albumin × −0.085), where bilirubin is in μmol/L and albumin in g/L.

Table S1 ALBI Score Classification and Corresponding Liver Dysfunction Severity

| ALBI Grade | ALBI Score | Description |
| --- | --- | --- |
| Grade 1 | ≤ −2.60 | mild liver dysfunction |
| Grade 2 | ＞ −2.60 to ≤ −1.39 | moderate liver dysfunction |
| Grade 3 | ＞ −1.39 | severe liver dysfunction |

# **Hematoxylin and Eosin staining**

Surgically resected tumor specimens were delivered to the Department of Pathology within 30 minutes after resection for formalin fixation and paraffin embedding, followed by sectioning into 4 μm-thick slices. After deparaffinization and rehydration through a graded series of alcohols , the sections were rinsed with distilled water. The sections were then stained with hematoxylin, followed by rinsing in running tap water to remove excess stain. The sections were differentiated in hydrochloric acid alcohol, then rinsed with water and blued in ammonia water. The slides were then stained with eosin and rinsed to remove excess dye. Following staining, the sections were dehydrated through an ascending ethanol gradient, followed by clearing in xylene. The sections were then mounted with neutral resin and covered with coverslips. All stained sections were examined under a microscope for evaluation of tissue morphology. All stained sections were independently observed and evaluated by a pathologist with 20 years of diagnostic experience in the digestive system.

**Edmonson-Steiner differentiation degree of tumor differentiation in hepatocellular carcinoma (HCC)**

- Well-differentiated: good differentiation, nuclear-to-cytoplasmic ratio close to normal, small tumor cell size, arranged in thin trabeculae.
- Moderately differentiated: larger cell size and higher nuclear-to-cytoplasmic ratio compared with well-differentiated, hyperchromatic nuclei, cellular atypia, eosinophilic granular cytoplasm, and occasional pseudo-glandular structures.
- Poorly differentiated: poor differentiation, larger cell size and higher nuclear-to-cytoplasmic ratio compared with moderately differentiated, marked cellular atypia, hyperchromatic nuclei, and frequent mitotic figures.
- Undifferentiated: least differentiated, scant cytoplasm, deeply stained nuclei, highly irregular cell morphology, poor adhesion, loose arrangement, and absence of trabecular architecture

# **Immunohistochemistry**

Formalin-fixed, paraffin-embedded tissue samples were cut into 4 μm sections. After deparaffinization and rehydration, antigen retrieval was performed. Endogenous peroxidase activity was quenched using 3% hydrogen peroxide, and nonspecific binding was minimized by incubation with 5% normal serum. Sections were then incubated overnight at 4 °C with primary antibodies at appropriate dilutions, followed by treatment with secondary antibodies and visualization using 3,3′-diaminobenzidine (DAB). After counterstaining with hematoxylin, the slides underwent differentiation in hydrochloric alcohol and were subsequently blued in ammonia water. Finally, sections were dehydrated and mounted for microscopic examination. All surgical specimens were independently observed and evaluated by a hepatopathologist with 20 years of diagnostic experience in the digestive system. The expression levels were semi-quantitatively scored on a scale from – (low expression) to +3 (high expression). Immunohistochemical analysis was conducted to evaluate the expression of GPC3 and Ki-67 in tumor tissues from both centers, while the expression of VEGFR2 and CK19 was additionally assessed in the Nanjing center.

**1.Glypican-3 (GPC3), Cytokeratin 19 (CK19) and Vascular Endothelial Growth Factor Receptor 2 (VEGFR2) Immunohistochemistry Scoring Criteria**

When GPC3, CK19 , VEGFR2 staining is positive, brown granules are observed in the cytoplasm, membrane, or lumen. The results are assessed based on both the proportion of positive cells and staining intensity. The scoring criteria for the proportion of positive cells are as follows:

- 0: 0-25%
- 1: 26-50%
- 2: 51-75%
- 3: >75%

Staining intensity is scored as follows:

- 0: No detectable staining (negative)
- 1: Light yellow staining (weak positive)
- 2: Brownish-yellow staining (moderate positive)
- 3: Dark brown staining (strong positive)

The final result is determined by multiplying the proportion score with the intensity score, which is then categorized into four levels:

- -: 0-1
- +: 2-4
- ++: 5-7
- +++: >8

**2. Ki-67 Immunohistochemistry Scoring**

Ki-67 positive staining is represented by brown nuclear granules. Tumor slides were observed under a 400× light microscope, and 500 tumor cells were counted to determine the number of Ki-67 positive cells. The percentage of Ki-67 positive cells was calculated by dividing the number of positive cells by the total number of cells counted (500 cells).

# **Supplementary Results**

**Feature selection by Recursive Feature Elimination (RFE)**

| Table S2 Accuracy with Varying Numbers of Variables Selected via REF | | |
| --- | --- | --- |
| Number of Variables | SVM Accuracy | RF Accuracy |
| 1 | 0.725 | 0.694 |
| 2 | 0.7185 | 0.643 |
| 3 | 0.7051 | 0.6895 |
| 4 | 0.7316 | 0.694 |
| 5 | 0.7228 | 0.7095 |
| 6 | 0.7228 | 0.7051 |
| 7 | 0.7295 | 0.7117 |
| 8 | 0.745 | 0.7228 |
| 9 | 0.7295 | 0.7206 |
| 10 | 0.7184 | 0.7228 |
| 11 | 0.7117 | 0.7405 |
| 12 | 0.7139 | 0.7317 |
| 13 | 0.7095 | 0.7183 |
| 14 | 0.714 | 0.725 |
| 15 | 0.7162 | 0.7339 |
| 16 | 0.7117 | 0.7162 |
| 17 | 0.7162 | 0.7228 |
| 18 | 0.7095 | 0.7339 |
| 19 | 0.7117 | 0.7272 |
| 20 | 0.6984 | 0.7339 |
| 21 | 0.7028 | 0.7383 |
| 22 | 0.7073 | 0.7383 |
| 23 | 0.7051 | 0.7405 |
| 24 | 0.7006 | 0.7427 |
| 25 | 0.6917 | 0.7361 |
| 26 | 0.7029 | 0.7272 |
| 27 | 0.7028 | 0.7316 |
| 28 | 0.6962 | 0.7383 |
| 29 | 0.6895 | 0.745 |
| 30 | 0.6917 | 0.7338 |

**Machine learning model configuration and hyperparameter tuning**
To ensure optimal performance, the hyperparameters of each machine learning model were selected based on 5-fold cross-validation:

**XGBoost:** The model was trained with parameters set as eta = 0.005, max_depth = 4, subsample = 0.5, colsample_bytree = 0.5, gamma = 0, and nrounds = 370.

**SVM:** A radial basis function (RBF) kernel was employed.

**KNN:** The optimal parameters were k = 45 and the triangular kernel function.

**Risk score distribution across MVI status**

Figure S1. HE-stained histological sections illustrating microvascular invasion (MVI) status: (A) M0, (B) M1, (C) M2.

| Table S3 Risk score distribution across MVI status | |
| --- | --- |
|  | risk score |
| M0 | 0.218 (0.182, 0.294) |
| M1 | 0.388 (0.291, 0.515) |
| M2 | 0.462 (0.341, 0.643) |

**Risk score distribution by histological grade**

Figure S2. HE-stained histological sections illustrating different degrees of tumor differentiation in hepatocellular carcinoma (HCC): (A) Well-differentiated, (B) Moderately differentiated, (C) Poorly differentiated, (D) Undifferentiated.

| Table S4 Risk score distribution by histological grade | |
| --- | --- |
|  | risk score |
| Well differentiated | 0.197 (0.174, 0.256) |
| Moderately differentiated | 0.236 (0.189, 0.345) |
| Poorly differentiated | 0.325 (0.229, 0.439) |
| Undifferentiated | 0.298 (0.257, 0.502) |

**Risk score distribution across GPC3, CK19 and VEGFR2 expression levels**

Figure S3. Immunohistochemical staining of Glypican-3 (GPC3) in hepatocellular carcinoma (HCC) tissues.
(A) GPC3 (–)
(B) GPC3 (+)
(C) GPC3 (++)
(D) GPC3 (+++)

| Table S5 Risk score distribution across GPC3, CK19 and VEGFR2 expression levels | | | |
| --- | --- | --- | --- |
|  | GPC3 | CK19 | VEGFR2 |
| - | 0.220 (0.183, 0.330) | 0.232 (0.188, 0.337) | 0.191 (0.164, 0.255) |
| ＋ | 0.240 (0.200, 0.333) | 0.320 (0.228, 0.418) | 0.241 (0.193, 0.356) |
| ＋＋ | 0.278 (0.217, 0.348) | 0.315 (0.252, 0.492) | 0.307 (0.222, 0.480) |
| ＋＋＋ | 0.319 (0.230, 0.404) | 0.313 (0.225, 0.501) | 0.291 (0.216, 0.365) |

# **Supplementary References**

1. Riley RD, Ensor J, Snell KIE, et al. Calculating the sample size required for developing a clinical prediction model. BMJ. 2020 Mar 18;368:m441. doi: 10.1136/bmj.m441.
2. Johnson PJ, Berhane S, Kagebayashi C, et al. Assessment of liver function in patients with hepatocellular carcinoma: a new evidence-based approach-the ALBI grade. J Clin Oncol. 2015 Feb 20;33(6):550-8. doi: 10.1200/JCO.2014.57.9151.
3. Cong WM, Bu H, Chen J, et al. Practice guidelines for the pathological diagnosis of primary liver cancer: 2015 update. World J Gastroenterol. 2016 Nov 14;22(42):9279-9287. doi: 10.3748/wjg.v22.i42.9279.
